# The impact of standardized care pathway on reporting time for invasive melanoma – results from one pathology department in Sweden

**DOI:** 10.1080/03009734.2019.1675102

**Published:** 2019-10-29

**Authors:** Margrét Agnarsdóttir, Helen Päären, Ismini Vassilaki

**Affiliations:** a Department of Clinical Pathology, Akademiska University Hospital, Uppsala, Sweden;; b Department of Immunology, Genetics and Pathology, Rudbeck Laboratory, Uppsala University, Uppsala, Sweden;; c Dermipath, Consulting Dermatopathology Service, Stockholm, Sweden

**Keywords:** Diagnosis, melanoma, pathology, quality, reporting time, standardized care pathway

## Abstract

**Background:** Standardized care pathway (SCP) was introduced by the Swedish health authorities to eliminate unwanted delay in the diagnostics of cancer patients; for melanoma, SCP started in 2016. The aim of this study was to investigate the impact of SCP on reporting time for invasive melanomas.

**Materials and methods:** Information on reporting time was collected on all samples handled according to the SCP and on all invasive melanomas diagnosed in 2016–2018 at the Department of Clinical Pathology, Akademiska University Hospital, Uppsala, Sweden.

**Results:** During the study period, 205 samples were handled according to the SCP, resulting in 53 cases (26%) diagnosed with invasive melanomas. A total of 301 invasive melanomas from 286 patients were diagnosed during the study period; 67 (22%) were submitted as SCP, 36 (12%) as a general priority case, and 198 (66%) as non-priority. The reporting time for the SCP cases was 8 days, for general priority cases 6 days, and for non-priority cases it was 24 days. The reporting time increased from 18 to 31 days for the non-priority cases and from 15 to 25 days for all cases with invasive melanomas during the study period.

**Conclusion:** This study demonstrates prolonged reporting times for invasive melanomas since the implementation of SCP. This is probably caused by the crowd-out effect of the SCP samples, limited personnel resources, and inaccuracy of the clinical diagnosis. SCP might therefore be a suboptimal method to shorten reporting times for invasive melanomas.

## Introduction

Invasive melanoma is the leading cause of skin-related deaths in the Western world (1,2). In Sweden, melanoma is the fifth most common cancer diagnosis in women and the sixth for men (3). It is important to diagnose these tumours at an early stage to secure good prognosis of the patients and to reduce waiting times – a significant cause of patients’ anxiety.

In many Swedish pathology departments, the reporting time for non-priority cases is long due to high workload, as there is a shortage of both pathologists and laboratory technicians. Traditionally, physicians are able to mark a case as priority on the pathology requisition form, resulting in a shorter reporting time, but at an additional cost. Standardized care pathway (SCP) was introduced by the Swedish health authorities to eliminate unwanted delay in the diagnostics of cancer patients; for melanoma, SCP started in May 2016. Similar programmes have been established in Denmark and Norway (4–6). According to the SCP clinical melanoma guidelines, it is important that the physician clearly writes on the pathology requisition form that the clinical diagnosis is melanoma, or high suspicion thereof, as SCP for skin lesions is only intended for melanomas. SCP is not to be used for ruling out melanomas or diagnosing other skin malignancies. For patients to be managed according to SCP, the pathology requisition is marked in a specific way. Therefore, these cases are treated as priority cases resulting in shorter reporting time but without any additional cost for the healthcare provider.

The aim of this study was to investigate the impact of SCP on reporting time for invasive melanomas.

## Materials and methods

All the cases were diagnosed at the Department of Clinical Pathology, Akademiska University Hospital, Uppsala, Sweden. The department provides clinical diagnostic services to other departments located at the university hospital (i.e. dermatology, plastic surgery, head and neck surgery, among others) and many general practitioner practices located outside of the hospital. In addition, it serves a small municipal hospital and a few private surgeons. The population of the area that the department serves was 360,124 in 2016 (7). During that year, the department received a total of 7701 dermatopathology cases, and all cases were taken care of by dermatopathologists.

### Patients with invasive melanoma

The patients included in the study were diagnosed with invasive melanoma during the years 2016 and 2017, in addition to the first half of the year 2018 (January until June). The patients were identified by a search through the laboratory information system (LIS) by using specific diagnostic SNOMED (Systematized Nomenclature of Medicine) codes. The following codes were used for the search: superficial spreading melanoma (SSM), M87433; nodular melanoma (NM), M87213; lentigo malignant melanoma (LMM), M87423; acral lentiginous melanoma (ALM), M87443; desmoplastic melanoma, M87453; and malignant melanoma unspecified, M87203.

The information registered for each patient was collected from the pathology requisition form, the pathology report, and from the LIS (Table 1). The glass slides were not reviewed. If the tumour thickness was reported as an interval, the higher number was registered as the thickness. If the tumour was biopsied and the remaining tumour later removed with an excision, information on the pathology requisition form coupled to the biopsy was registered. During the years 2016 and 2017, the T-stage was registered according to the American Joint Committee of Cancer (AJCC), 7th edition 2009 (8). In 2018, the T-stage was registered according to the updated 8th edition 2017 (9). The reporting time was defined as the time in calendar days between the date of arrival of the specimen to the pathology department and the date of signature of the electronic report by the pathologist. Patients with pathology requisition forms marked as SCP but with a form not filled out correctly according to guidelines and therefore not treated as SCP were included among the non-priority cases (*n* = 12). Forms where the case was marked with both SCP and as a general priority case were included among the SCP cases (*n* = 12). Cases where SCP was missed – at the Department of Clinical Pathology, or the clinicians failed to mark the pathology form (information found in the LIS) – were included among the non-priority cases (*n* = 6). For the clinical diagnosis found on the pathology requisition form, the diagnoses were divided into the following groups: (a) melanoma or high suspicion thereof; (b) different melanocytic lesions (including benign naevus, dysplastic naevus, *in situ* and invasive melanomas); (c) various different tumour types (e.g. melanoma/seborrhoeic keratosis, basal cell carcinoma/melanoma); (d) basal cell carcinoma; (e) benign lesions (e.g. seborrhoeic keratosis, pyogenic granuloma, hemangioma); and (f) other, i.e. no specific diagnosis revealed on the pathology requisition form.

**Table 1.** Information collected for each patient/tumour.

Patient/tumour details collected
Gender
Age at diagnosis
Tumour localization
Case marked with priority (SCP or a general priority case)
Healthcare provider
Clinical diagnosis/question on pathology requisition form
Surgical excision or biopsy material
Reporting time in calendar days
Additional glass slides generated at the histopathology laboratory
Immunohistochemistry performed
Subtype (SSM, NM, LMM, other)
T-stage found in pathology report
Tumour thickness in mm
Pre-existing melanocytic naevus
Discussed at a multidisciplinary meeting
The pathology form for the melanoma quality register completed

LMM: lentigo malignant melanoma; NM: nodular melanoma; SCP: standardized care pathway; SSM: superficial spreading melanoma.

### Patients diagnosed with invasive melanomas in the year 2015 (a comparison group)

This group was identified with a search in the LIS employing the same SNOMED codes as for the patients diagnosed in the years 2016–2018 (see above). For the patients diagnosed in the year 2015, the only information collected was the reporting time and whether or not the pathology requisition form was marked with priority. In the year 2015 SCP had not been introduced.

### Patients handled according to the SCP

In the LIS all patients handled according to the SCP are marked in a specific way that is independent of the diagnostic SNOMED code. To identify the patients a search was carried out in the LIS during the included time period (2.5 years), and the diagnosis for each patient was registered.

### Statistics

The results are presented as descriptive statistics in the form of absolute numbers and percentage; median and average calculations were performed in Microsoft Excel.

### Ethical statement

The Uppsala Regional Ethical Review Board was consulted, and they had no ethical objections for the implementation of the study (Ref. 2017/438).

## Results

### Patients handled according to the SCP

During the study period, a total of 205 cases were handled according to the SCP at the Department of Clinical Pathology. Fifty-three cases (25.9%) were diagnosed as invasive melanomas and 40 as *in situ* melanomas (19.5%). The remaining cases represented various other melanocytic lesions (*n* = 63), benign non-melanocytic lesions (*n* = 29), basal cell carcinoma (*n* = 10), and *in situ* squamous cell carcinoma (*n* = 2). The majority of the SCP cases with the histopathological diagnosis of invasive melanoma (*n* = 53) were submitted from hospital departments (Department of Dermatology, *n* = 17; other departments, *n* = 27).

### Patients diagnosed with invasive melanomas

During the study period, a total of 301 invasive melanomas from 286 patients were diagnosed at the Department of Clinical Pathology. The median age was 67 years (SD 15). The cases were evenly distributed between men and women, the most common location was the trunk, and the dominant morphological subtype was SSM when stated (Table 2).

**Table 2.** Demographic and clinical data for the invasive melanomas diagnosed in 2016–2018 (*n* = 301).

Demographic and clinical data	Number (%)
Healthcare provider	
Department of dermatology	79 (26.2)
Other hospital departments	67 (22.3)
General practitioners	105 (34.9)
Others outside of the hospital	50 (16.6)
Clinical diagnosis on pathology form	
Melanoma	114 (37.9)
DDx melanocytic lesion	55 (18.3)
Various tumour types	51 (16.9)
Basal cell carcinoma	10 (3.3)
Benign lesion	7 (2.3)
No specific diagnosis	64 (21.3)
Gender	
Men	151 (50.2)
Women	150 (49.8)
Treatment	
Local excision	239 (79.4)
Biopsy	62 (20.6)
Location	
Trunk	170 (56.5)
Lower extremity	48 (16.0)
Upper extremity	47 (15.6)
Head and neck	16 (5.3)
Face	18 (6.0)
Ear	1 (0.3)
Missing information	1 (0.3)
Morphological subtype	
SSM	126 (41.9)
LMM	16 (5.3)
NM	14 (4.7)
Other types	9 (3.0)
Not stated	136 (45.1)
T-stage	
pT1	194 (64.5)
pT2	48 (15.9)
pT3	25 (8.3)
pT4	29 (9.6)
pTx	5 (1.7)

DDx: differential diagnosis; LMM: lentigo malignant melanoma; NM: nodular melanoma; SSM: superficial spreading melanoma.

The clinical diagnosis of melanoma or the differential diagnosis of a melanocytic lesion was only found on half of the pathology forms (Table 2). These clinical diagnoses were more often noted on pathology requisition forms in the year 2018 (*n* = 37; 63.8%) compared with the year 2016 (*n* = 60; 51.8%). The clinical diagnosis of melanoma was more often noted on pathology requisition forms submitted from departments located at the hospital (*n* = 82/114; 72%; Department of Dermatology *n* = 35; other departments *n* = 47) compared with healthcare providers outside of the hospital (*n* = 32/114; 28.0%; general practitioners *n* = 17; others *n* = 15).

For the cases where the clinical diagnosis was melanoma or high suspicion thereof (*n* = 114), only nine were biopsied (Table 2). Among these 114 cases, 76 were marked as priority cases, either according to SCP or as general priority cases.

During the study period, there was an increase in the number of pathology reports where the subtype was stated by the dermatopathologist (2016: *n* = 40, 34.5%; 2017: *n* = 75, 59.1%; 2018: *n* = 50, 86.2%).

The median thickness of the tumours was 0.7 mm, and the majority were thin melanomas, pT1a and pT1b (Table 2). The majority of the pT1 tumours were from women (*n* = 103; 34.2%) compared with men (*n* = 91; 30.2%). As for the pT2–pT4 tumours, the majority were from men (*n* = 57; 18.9%) compared with women (*n* = 45; 15.0%). Information on T-stage was found in 91.4% (*n* = 275) of the pathology reports.

According to the pathology reports, 109 tumours (36.2%) originated from a pre-existing naevus. The majority of the patients were discussed at a multidisciplinary meeting (*n* = 265; 88.7%). The pathology form for the melanoma quality register in Sweden was completed for the vast majority of the cases (*n* = 295; 98.0%).

### Reporting time of the patients with invasive melanoma and the application of SCP

The median reporting time during the study period was 8 days for the SCP cases, 6 days for the general priority cases, and 24 days for the non-priority cases. The reporting time increased from 18 to 31 days for the non-priority cases and from 15 to 25 days for all cases with invasive melanomas during the study period (Table 3). For comparison, in the year 2015, before SCP was introduced, the reporting time for priority cases was 6 days, for non-priority cases 26 days, and for all cases 21 days.

**Table 3.** The reporting time for the invasive melanoma cases in the different priority groups including the standardized care pathway (SCP). The number of cases in each group and the healthcare provider submitting the SCP cases (percentage in parentheses).

	2015^a^	2016	2017	2018	2016–2018
Reporting time					
All cases	21	15	20	25	18
SCP		7	10	6	8
General priority	6	5	7	6	6
SCP and priority		5	9	6	7
Non-priority	26	18	25	31	24
Number					
SCP		14 (12.0)	34 (26.8)	19 (32.8)	67 (22.2)
General priority	28 (24.8)	22 (19.0)	11 (8.7)	3 (5.2)	36 (12.0)
Non-priority	85 (75.2)	77 (66.4)	75 (59.0)	28 (48.3)	180 (59.8)
Others^b^		3 (2.6)	7 (5.5)	8 (13.7)	18 (6.0)
Total	113	116	127	58	301
Healthcare provider SCP					
Department of dermatology		4 (6.0)	13 (19.4)	5 (7.4)	22 (32.8)
Other hospital departments		8 (11.9)	14 (21.0)	8 (11.9)	30 (44.8)
General practitioners		2 (3.0)	6 (9.0)	4 (6.0)	12 (18.0)
Others outside of the hospital		0	1 (1.5)	2 (3.0)	3 (4.5)
Total		14	34	19	67

^a^SCP not implemented.

^b^Cases where SCP or priority was missed or the pathology form not correctly filled out and therefore not handled as SCP.

There was a gradual increase in the number of SCP cases during the study period, with a corresponding decrease in the number of general priority cases. Hospital departments were more prone to use SCP than other healthcare providers (Table 3).

The majority of the SCP cases were pT1 tumours (Table 4); among the thick melanomas pT4, only 12 out of 29 cases (41.4%) were marked with priority (SCP *n* = 9; general priority *n* = 3).

**Table 4.** T-Stage of the standardized care pathway (SCP) cases and the healthcare providers submitting the cases.

	pT1	pT2	pT3	pT4	Total
Department of dermatology	17	2	2	1	22
Other hospital departments	17	4	2	7	30
General practitioners	10	1	0	1	12
Others outside of the hospital	2	1	0	0	3
Total	46	8	4	9	67

### Additional laboratory procedures and the effect on reporting time

For 107 cases (35.5%), more slides were generated at the histopathology laboratory before signing out the case, mostly deeper sections. For 123 cases (40.9%), immunohistochemistry was added. For 62 cases (20.6%), both applied. If additional laboratory procedures were added the reporting time during the study period increased by 4 days for the SCP cases and by 3 days for the general priority cases. For the non-priority cases the reporting time increased by 4 days.

## Discussion

This study demonstrates prolonged reporting times for invasive melanomas since the implementation of SCP at a pathology department located at a university hospital in Sweden.

During the study period, the median reporting time for all cases was long, 18 calendar days. For cases handled with priority (general priority case and SCP), the reporting time was in the range of 5–9 days, which is within the SCP’s recommended time period. According to SCP, the recommended time period from excision to information to the patient is 14 days. For non-priority cases, the reporting time gradually became longer during the study period, from 18 days in 2016 to 31 days in 2018. The variation in the reporting time observed for the non-priority cases is related to high workload, shortage of both dermatopathologists and laboratory technicians, and the possible crowd-out effect of the SCP samples. In addition, inaccuracy in the clinical diagnosis resulted in more than half of the melanomas were underdiagnosed clinically and were found among the non-priority cases.

The SCP for melanoma started formally in May 2016, but the first case was received at the Department of Clinical Pathology in the end of March 2016. However, problems were observed with implementation of this new routine at the department, e.g. other tumour types being marked as SCP, pathology forms not filled out correctly, cases marked both with SCP and also as a general priority case. This resulted in problems with implementation and handling at the department. However, during the study period there was a gradual increase in the number of cases handled according to the SCP. Still, it is of concern that during the first half of 2018, when SCP had been implemented for 2 years, half of the invasive melanomas were submitted with no priority at all, including both general priority and SCP. It is also of concern that among the cases that were handled according to the SCP during the study period, less than half were actually melanomas, either *in situ* or invasive.

The drawback of this study is that we have not found similar studies to compare our results with, and the study includes only the first years after the implementation of SCP. The strength of the study is the systematic review of the cases found at one pathology department where all the cases were taken care of by dermatopathologists, not general pathologists.

The pathway for patients undergoing diagnosis and treatment for melanoma is complex and involves many factors. This study reveals that despite efforts to shorten the reporting time for melanomas, the introduction of the SCP has not been successful, as the majority were not submitted as a SCP case, and the reporting time for the non-priority cases – the group where the vast majority of the melanomas was found – increased. We can only speculate why the SCP is not more successful in lowering the reporting time during the included study period. The number of physicians seeing this patient group is vast; therefore, adequate and repeated information about this new routine and its consequences is very important. The clinical experience varies among treating physicians, as does the clinical appearance of this tumour type, making it difficult to diagnose, even for experienced dermatologists. This study also demonstrates the possible crowd-out effect of the SCP cases on the reporting time of non-priority cases at a pathology department with limited personnel resources, when a subgroup of cases is handled with priority.

Perhaps the optimal solution would have been to invest funding, time, and effort to support the pathology departments in order to facilitate decreased lead times during the complex laboratory and diagnostic processes.

In conclusion, this study demonstrates prolonged reporting times for invasive melanomas since the implementation of SCP. This is probably caused by the crowd-out effect of the SCP samples, limited personnel resources, and inaccuracy of the clinical diagnosis. SCP might therefore be a suboptimal method to shorten reporting times for invasive melanomas, but reports and studies from other departments are also necessary to evaluate the long-term effect.
